# Sex-Specific Alterations of the Lung Transcriptome at Birth in Mouse Offspring Prenatally Exposed to Vanilla-Flavored E-Cigarette Aerosols and Enhanced Susceptibility to Asthma

**DOI:** 10.3390/ijerph20043710

**Published:** 2023-02-20

**Authors:** Alexandra Noël, Sultan Yilmaz, Tori Farrow, Matthew Schexnayder, Oliver Eickelberg, Tomislav Jelesijevic

**Affiliations:** 1 Department of Comparative Biomedical Sciences, School of Veterinary Medicine, Louisiana State University, Baton Rouge, LA 70803, USA; 2 Department of Environmental Toxicology, Southern University and A & M College, Baton Rouge, LA 70813, USA; 3 IDEXX Laboratories, Inc., One IDEXX Drive, Westbrook, ME 04092, USA; 4 Division of Pulmonary, Allergy, and Critical Care Medicine, Department of Medicine, University of Pittsburgh, Pittsburgh, PA 15261, USA

**Keywords:** electronic nicotine delivery system (ENDS), electronic cigarette, in utero exposures, prenatal exposures, asthma, developmental origins of health and disease

## Abstract

Currently, approximately 8 million adult Americans use electronic cigarettes (e-cigs) daily, including women of childbearing age. It is known that more than 10% of women smoke during their pregnancy, and recent surveys show that rates of maternal vaping are similar to rates of maternal cigarette smoking. However, the effects of inhaling e-cig aerosol on the health of fetuses remain unknown. The objective of the present study was to increase our understanding of the molecular effects caused by in utero exposures to e-cig aerosols on developing mouse lungs and, later in life, on the offspring’s susceptibility to developing asthma. Methods: Pregnant mice were exposed throughout gestation to either filtered air or vanilla-flavored e-cig aerosols containing 18 mg/mL of nicotine. Male and female exposed mouse offspring were sacrificed at birth, and then the lung transcriptome was evaluated. Additionally, once sub-groups of male offspring mice reached 4 weeks of age, they were challenged with house dust mites (HDMs) for 3 weeks to assess asthmatic responses. Results: The lung transcriptomic responses of the mouse offspring at birth showed that in utero vanilla-flavored e-cig aerosol exposure significantly regulated 88 genes in males (62 genes were up-regulated and 26 genes were down-regulated), and 65 genes were significantly regulated in females (17 genes were up-regulated and 48 genes were down-regulated). Gene network analyses revealed that in utero e-cig aerosol exposure affected canonical pathways associated with CD28 signaling in T helper cells, the role of NFAT in the regulation of immune responses, and phospholipase C signaling in males, whereas the dysregulated genes in the female offspring were associated with NRF2-mediated oxidative stress responses. Moreover, we found that in utero exposures to vanilla-flavored e-cig aerosol exacerbated HDM-induced asthma in 7-week-old male mouse offspring compared to respective in utero air + HDM controls. Conclusions: Overall, these data demonstrate that in utero e-cig aerosol exposure alters the developing mouse lung transcriptome at birth in a sex-specific manner and provide evidence that the inhalation of e-cig aerosols is detrimental to the respiratory health of offspring by increasing the offspring’ susceptibility to developing lung diseases later in life.

## 1. Introduction

Currently, approximately 8 million adult Americans, including women of childbearing age, use electronic nicotine delivery systems (ENDSs) on a regular basis, which are also known as electronic cigarettes (e-cigs) [1]. Since ENDSs deliver nicotine without combustion, they are often perceived by the general public as ‘safer’ alternatives to conventional cigarettes [2]. Research published from 2007 to 2017 shows that up to 15% of pregnant women in the United States use ENDSs [3]. These prevalence findings on vaping during pregnancy are alarming because there continues to be a lack of reliable information regarding the risks of using ENDSs on maternal and fetal health. Maternal smoking is considered the primary preventable factor in several pregnancy-related morbidity and mortality outcomes [4]. As shown from studies on active smoking during pregnancy, nicotine is one of the most important agents in conventional cigarette smoke that is responsible for altering prenatal development. Nicotine is a known teratogen, which, as a lipophilic molecule, readily crosses the placenta and accumulates in placental tissue, amniotic fluid, and fetal serum [3,5]. Since e-cigs are marketed mainly as nicotine delivery devices, it is vitally important to investigate the effects of vaping during pregnancy on the developing lungs of the fetus. In addition to nicotine, e-cig aerosols contain other toxicants, including aldehydes (e.g., formaldehyde, acetaldehyde, and acrolein) and flavoring chemicals, which further contribute to the toxicity of e-cig aerosols. Thus far, exclusive ENDSs use during pregnancy has been shown to cause adverse outcomes similar to those observed with maternal smoking [6,7,8]. Three independent studies [6,7,8] have analyzed data collected in the Pregnancy Risk Assessment Monitoring System (PRAMS) cohorts and demonstrated that exclusive ENDS use during pregnancy can cause adverse pregnancy outcomes, including pre-term birth (adjusted prevalence ratio [PR] = 1.69; OR = 1.86), low birth weight (PR = 1.52–1.88; OR = 1.53), and infants born small for gestational age (OR = 1.76); these measures of association were all significant when compared with non-tobacco-using pregnant women [6,7,8]. Fetal growth restriction represents a clinical indicator associated with increased risk for severe complications in neonatal morbidities, including lung diseases [9,10]. Thus, these human data clearly show that vaping during pregnancy is associated with adverse birth effects, harming maternal and child health. This highlights that neonatal morbidity from gestational exposure to ENDS aerosols is a preventable outcome of great importance. Overall, the use of ENDSs during pregnancy is a major public health issue that urgently needs to be addressed via additional epidemiological and experimental studies.

Numerous epidemiological and experimental studies have demonstrated that in utero exposure to cigarette smoke, second-hand smoke, or nicotine are important risk factors for asthma development in offspring [11,12,13]. As noted above, e-cig aerosol composition differs from that of cigarette smoke or second-hand smoke, as it includes heated and aerosolized flavorings, propylene glycol (PG), and glycerin (G). This creates a clear need for a direct evaluation of the in utero effects of e-cig aerosols on fetal lung development and on the developmental origins of health and disease. This concept, also known as the Barker hypothesis, suggests that risk factors for the intrauterine environment can affect fetal development, which increases the risk for chronic diseases later in life [14]. Lung organogenesis is a window of development particularly sensitive to environmental insults [15,16,17,18]. Lung development is a process that requires highly specific temporal and dimensional control of gene and protein expression in order to attain a functional lung architecture at birth [16,17]. Maternal exposures to environmental pollutants, including particulate matter and second-hand smoke, can directly impact the stability of the fetal lung transcriptome [19,20,21]. Further, in utero exposures to ENDS aerosols, with or without nicotine, can induce transcriptomic changes in the brain and lung tissues of mice [22,23,24,25,26]. Still, the underlying molecular mechanisms associated with the in utero ENDS aerosol exposure-induced lung transcriptomic alterations and how these effects subsequently affect lung physiology in childhood and adulthood, are still poorly understood [17].

Studies using rats have shown that in utero exposure to nicotine induces epigenetic alterations, evidenced by histone acetylation in the lung tissue, which resulted in an asthmatic phenotype in the offspring [27]. Another study demonstrated that in utero exposures to e-cig aerosols caused global DNA methylation in mice offspring [9]. These two studies indicate that in utero nicotine and e-cig aerosol exposures induce epigenetic modifications, potentially leading to an increased risk of developing asthma. Furthermore, we previously showed that mouse offspring exposed in utero to cinnamon-flavored e-cig aerosols containing 36 mg/mL of nicotine exhibited altered *Wnt* signaling in the lungs and an impaired expression of epigenetic chromatin modification genes at birth and at 5 days of age, respectively [22,28]. Additionally, we recently showed that in utero exposures to mint-flavored JUUL aerosols containing 5% nicotine salt exacerbated house dust mite (HDM)-induced asthmatic responses in 11-week-old mouse offspring [23]. Taken together, these studies suggest that in utero exposures to nicotine or e-cig aerosols affect normal fetal lung development and pose a risk for the development of lung diseases, including asthma. Since maternal vaping is increasing [3] and asthma treatment is an unmet medical need, it is vitally important to investigate the molecular effects caused by in utero e-cig exposure on the lungs and how this potentially predisposes to asthma later in life. The overall goal of this study was to increase our understanding of the molecular effects caused by in utero exposures to e-cig aerosols on developing mouse lungs and, later in life, on the offspring’s susceptibility to developing asthma. We selected vanillin, an e-cig flavoring chemical known to cause DNA damage [29,30], to evaluate how in utero exposures of mice to vanilla-flavored e-cig aerosols containing 18 mg/mL of nicotine affected the lung transcriptome at birth and then aggravated asthmatic responses following HDM in 7-week-old male mouse offspring. This study provides scientific evidence showing that inhalation of e-cig aerosols is not a “safe” alternative to conventional cigarettes in the context of pregnancy, particularly regarding the respiratory health of the offspring.

## 2. Methods

### 2.1. Overall Study Design

Pregnant mice were exposed for 20 consecutive days during gestation to either filtered air or vanilla-flavored e-cig aerosols containing 18 mg/mL of nicotine. Sub-groups of male and female in utero-exposed mouse offspring were sacrificed at birth, and the lung transcriptome was analyzed. When additional in utero-exposed male mouse offspring reached 4 weeks of age, they were treated with house dust mites (HDMs) or saline and sacrificed at 7 weeks of age for the assessment of lung responses.

### 2.2. ENDS Aerosol Generation and Characterization

The vanilla-flavored e-liquid containing 18 mg/mL of nicotine (50/50 PG/G) was purchased online from EC Blend (Medford, OR, USA). We used a third-generation e-cig device, a Joyetech eVic VTC mini mod, which was connected to a SCIREQ e-cigarette aerosol generator (Scireq^®^, Montreal, QC, Canada), to produce the e-cig aerosols, as previously described [31]. We followed the CORESTA standards [32] to generate the e-cig aerosols (1 puff every 30 s and a 55 mL puff volume). The e-cig exposure system was connected to a 5 L whole-body exposure chamber (InExpose, Scireq^®^, Montreal, QC, Canada) in which the mice were exposed to the vanilla-flavored e-cig aerosols. Concomitantly, the control mice were exposed to filtered air in a similar chamber. We conducted real-time and offline characterization of the e-cig aerosols via a MicroDustPro (Casella) and traditional gravimetric sampling methods, respectively, as previously described [22,28]. The chemical analysis of the vanilla-flavored e-cig aerosol produced under the same conditions as those in this study was published by our research group elsewhere [33] and revealed that this vanilla-flavored e-cig aerosol contained high concentrations of PG and G (>2 mg/puff) in addition to having elevated levels of butyraldehyde (>1 µg/puff).

### 2.3. Animal Exposures

For this in vivo study, we used pregnant 12-week-old female BALB/c mice (Charles River, Wilmington, MA, USA). These mice were exposed in two separate sessions to either filtered air or vanilla-flavored ENDS aerosols for 2 h per day for 20 consecutive days during gestation. The exposures of the pregnant mice to ENDS aerosols were conducted as previously described [22,23,28]. In total, 15 dams exposed to air and 9 dams exposed to vanilla-flavored e-cig aerosol gave birth, with similar average litter sizes of 6.93 and 6.44 pups, respectively. The average birth weight of the pups was also similar, with 1.39 g and 1.40 g for the pups exposed in utero to air and e-cig aerosol, respectively. We controlled for ‘litter effects’ by using one offspring per sex per litter for the biological endpoint analyzed. At birth, sub-groups of mouse offspring were euthanized by using an intraperitoneal injection of Beuthanasia-D (Schering-Plough, Kenilworth, NJ, USA), followed by decapitation. The lungs of those 1-day-old offspring were excised and stored in RNAlater, for subsequent RNA sequencing analysis. After weaning (4 weeks of age), additional sub-groups of male mouse offspring were treated with intranasal instillation of either HDM in order to induce asthmatic responses or saline for the control treatment. Intranasal instillations were carried out once a week for 3 consecutive weeks. At 7 weeks of age, following the completion of the HDM or saline treatment, the male offspring were euthanized by using an intraperitoneal injection of Beuthanasia-D (Schering-Plough, NJ, USA). The samples were collected and stored for subsequent analyses. All of the mice were housed in an AAALAC-approved animal care facility at the Louisiana State University School of Veterinary Medicine under a 12 h light/dark cycle (from 6:00 a.m. to 6:00 p.m.). The mice had access to water and food ad libitum, except during the 2 h exposure periods. The mice were housed and handled in accordance with the NIH Guide for the Care and Use of Laboratory Animals. All of the procedures and protocols were approved by the Louisiana State University Institutional Animal Care and Use Committee.

### 2.4. House Dust Mite Treatment

We purchased HDMs from Stallergenes Greer (Lenoir, NC, USA) (D. pteronyssinus, catalogue number: XPB70DA2.5). The HDM extract was resuspended in saline. The HDM treatment was initiated when the in utero-exposed male mouse offspring were 4 weeks of age. The mice were first anesthetized, and then, 10 μL per nostril of HDM or saline was administered through intranasal instillation. Each HDM-treated mouse received 25 μg of HDM. This HDM treatment was administered once a week for three consecutive weeks.

### 2.5. Lung Function—Whole-Body Plethysmography

We used a whole-body plethysmograph (Buxco, Troy, NY, USA) to measure tidal volume in 7-week-old male mice in utero-exposed offspring non-invasively, as previously described [34]. This lung function parameter was measured once per minute for 5 min.

### 2.6. Bronchoalveolar Lavage Fluid (BALF)

At 7 weeks of age, the in utero-exposed male mouse offspring were euthanized, and BALF was collected. The lungs were lavaged twice with 0.5 mL of saline. The collected BALF was subsequently processed using a cytospin to prepare the slides, as previously described [23]. The slides with the cells were then stained with a modified Wright’s stain. The BALF differentials were assessed on 300 cells by a board-certified clinical pathologist who was blinded to the treatments.

### 2.7. Histopathological Evaluation of Lung Tissue

After the BALF collection from the 7-week-old male mouse offspring, the lungs were pressured-fixed and inflated with 10% formalin. The tissues were then processed for standard hematoxylin and eosin (H&E) and periodic acid-Schiff (PAS) staining. The histopathology evaluation of the lung tissue included lung peribronchial and perivascular inflammation, as well as the mucous metaplasia of the airways. All of the parameters were scored according to an arbitrary scale of 0 to 5, where 0 = almost none or none; 0.5 = minimal spotty; 1 = minimal evenly distributed; 2 = mild (low); 3 = moderate; 4 = severe (high); and 5 = bronchial cuffs. Histopathological evaluation of the lung tissues was performed by a board-certified veterinary pathologist who was blinded to the treatments.

### 2.8. RNA Extraction

The RNA was isolated from the mouse lung tissues using the Qiagen RNeasy Mini Kit (catalog number 74104) with Trizol/chloroform extraction, and the RNA concentration and purity were determined with a NanoDrop ND-1000 Spectrophotometer (NanoDrop, Wilmington, DE, USA).

### 2.9. RNA Sequencing

We evaluated the lung transcriptome profiling of lung homogenates from 1-day-old in utero-exposed male and female mouse offspring (n = 4 per group) via RNA sequencing (poly A). Following the RNA extraction of the lung samples, the RNA was shipped to LC Sciences (Houston, TX, USA) to be further processed and sequenced. A poly (A) RNA sequencing library was prepared by LC Sciences following Illumina’s TruSeq-stranded-mRNA sample preparation protocol. The integrity of the RNA was verified with an Agilent Technologies 2100 Bioanalyzer. Quality control analysis and the quantification of the sequencing library were performed using Agilent Technologies 2100 Bioanalyzer High Sensitivity DNA Chip. Paired-ended sequencing was performed on Illumina’s NovaSeq 6000 sequencing system. The sequencing depth was 150 bp paired-end, >40 million reads. Bioinformatics analyses were subsequently performed by LC Sciences. The differentially expressed mRNA were selected with a log2 |fold-change| > 1.5 and with a statistical significance of *p* < 0.05.

### 2.10. RT2 PCR Arrays

As previously described in Cahill et al. [23], we used the RT2 First Strand Kit (Qiagen 330401) and followed the manufacturer’s instructions to prepare cDNA from the extracted lung RNA of the 7-week-old in utero-exposed male mouse offspring. The cDNA was diluted to the required concentration with high-quality RNase-free water. The cDNA was then combined with the RT2 SYBR Green qPCR Master mix (Qiagen 330503). The resulting samples were used to assess the expression of 84 genes related to allergy and asthma (Catalog number 330,231 PAMM-067Z; RT^2^ Profiler™ PCR Array Mouse Allergy & Asthma) by profiler PCR arrays (Qiagen, Germantown, MD, USA), which were run according to the manufacturer’s instructions on an Applied Biosystems 7300 Real-Time PCR System. The results were expressed as threshold cycle (Ct) values. We used the Qiagen GeneGlobe data analysis software (RT^2^ Profiler PCR Array Data Analysis V 3.5) to calculate for each gene—via the ΔΔCt method—the fold-changes of the treatment groups compared to the air + saline control group.

### 2.11. Ingenuity Pathway Analysis (IPA)

The gene expression results obtained from the lungs of the 1-day-old in utero-exposed mouse offspring were analyzed using Qiagen’s Ingenuity Pathway Analysis (IPA). This allowed the identification of the canonical pathways associated with the dysregulated genes.

### 2.12. Statistical Analyses

We performed the statistical analyses using GraphPad Prism software (version 9.5.0). The results are reported as mean ± standard error of the mean (SEM). We used a one-way analysis of variance (ANOVA) (3 groups or more) followed by Tukey’s post hoc test to test whether there were any statistically significant differences between the groups. We examined the histopathology data, expressed as median ± interquartile range, via the Kruskal–Wallis test to determine the statistical differences between the groups. The results were considered statistically significant with a *p*-value of <0.05 or a |fold-change|of >1.5 for the PCR array gene expression data.

## 3. Results

### 3.1. In Utero Exposures to Vanilla-Flavored E-Cig Aerosols Significantly Alter the Lung Transcriptome of Newborn Male and Female Mice

**Figure 1:**
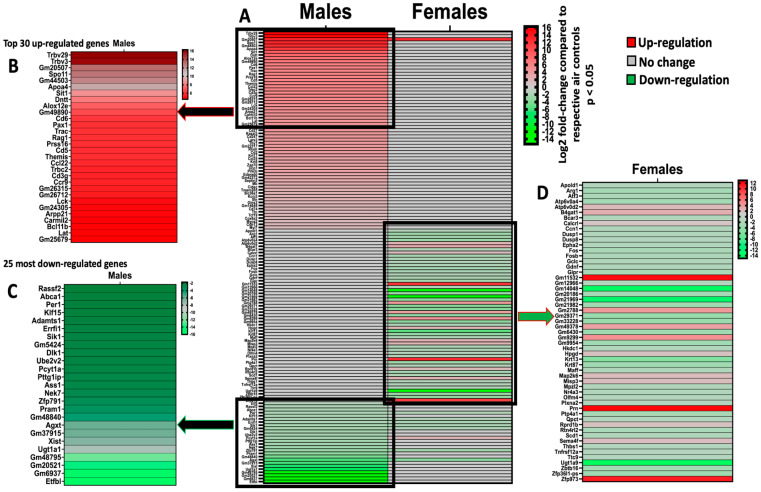
Prenatal exposures to vanilla-flavored e-cig aerosols alter the developing mouse lung transcriptome at birth. The significantly dysregulated genes obtained through RNA sequencing in the lungs of male and female mouse offspring at birth. (**A**) Heatmap of all significantly dysregulated genes in both male (88 genes) and female (65 genes) mouse offspring exposed in utero to vanilla-flavored e-cig aerosol compared to respective air controls. (**B**) Insert displaying the top 30 most up-regulated genes in male mouse offspring. (**C**) Insert displaying the 25 most down-regulated genes in male mouse offspring. (**D**) Insert displaying 55 dysregulated genes in female mouse offspring. Red = up-regulation; green = down-regulation. Values are presented as fold-changes compared to respective air controls. All genes displayed are expressed as Log_2_ |fold-change| > 1.5 with a *p*-value < 0.05. N = 4 per group.

**Figure 2:**
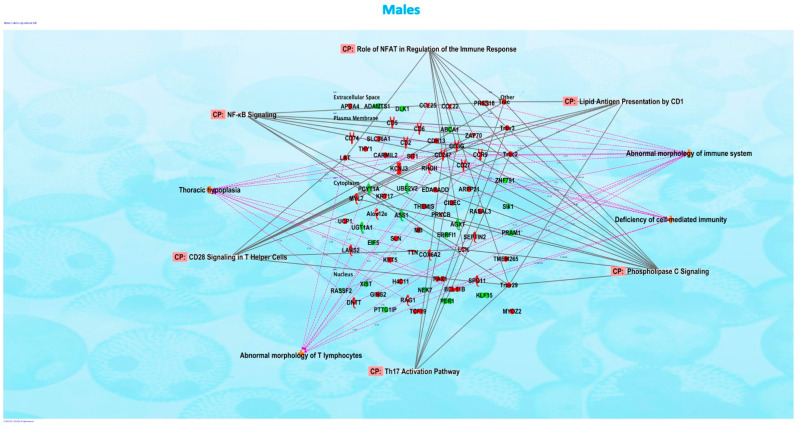
Ingenuity Pathway Analysis revealed that networks of inter-connected genes for protein-coding genes were associated with CD28 signaling in T-helper cells, Th17 activation pathway, and phospholipase C signaling in the male offspring mice exposed in utero to vanilla-flavored e-cig aerosol and sacrificed at birth.

**Figure 3:**
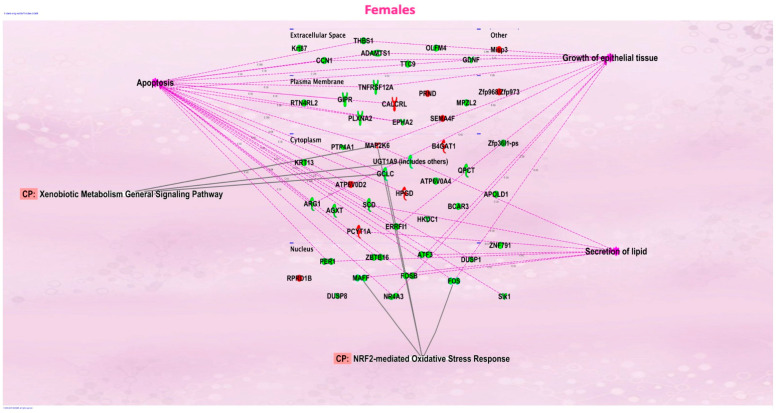
Ingenuity Pathway Analysis revealed that networks of inter-connected genes for protein-coding genes were associated with NRF2-mediated oxidative stress responses in the female offspring mice exposed in utero to vanilla-flavored e-cig aerosol and sacrificed at birth.

We evaluated the effects of in utero exposure to vanilla-flavored e-cig aerosols on newborn male and female mouse offspring lung transcriptomes. Lung organogenesis is a biological process highly sensitive to environmental toxicants [15,17,18], including in utero exposure to e-cig aerosols [10,22,23,28]. The e-cig aerosol exposure characterization results for our pregnant mice are shown in Table 1. The RNA-sequencing lung transcriptomic data of mouse offspring at birth (|fold-change| > 1.5 and *p* < 0.05) showed that the in utero vanilla-flavored e-cig aerosol exposure significantly dysregulated 88 genes in male offspring (62 genes were up-regulated and 26 genes were down-regulated) compared to the in utero air-exposed males. Sixty-five genes were significantly dysregulated in female offspring exposed in utero to vanilla-flavored e-cig aerosol (17 genes were up-regulated and 48 genes were down-regulated) when compared to the in utero air-exposed females (Figure 1). These dysregulated genes include protein-coding genes and long intergenic noncoding RNA (lincRNA), e.g., Gm20186, which are RNA transcripts larger than 200 nucleotides [35]. Up-regulated genes in the in utero vanilla-flavored e-cig aerosol males included *Cd5*, *Cd6*, *Ccl22*, and *Ccr9*, four protein-coding genes involved in T-cell immune responses and chemokine signaling, while *Map2k6* is a protein-coding gene induced by environmental stress [36], and *B4gat1* is involved in protein glycosylation, including on extracellular membrane proteins [37,38], which were up-regulated in the females. Ingenuity Pathway Analysis (IPA) revealed that in utero e-cig aerosol exposure affected the canonical pathways associated with CD28 signaling in T-helper cells, the role of NFAT in the regulation of immune responses, and phospholipase C signaling in males (Figure 2), whereas the dysregulated genes in the female offspring were associated with NRF2-mediated oxidative stress responses (Figure 3). Together, these data demonstrate that transplacental exposures to vanilla-flavored e-cig aerosols can disrupt gene expression in developing mouse lungs and that these molecular effects are sex-specific.

### 3.2. In Utero Exposures to Vanilla-Flavored E-Cig Aerosols Significantly Aggravate HDM-Induced Asthmatic Responses in 7-Week-Old Male Mouse Offspring

**Figure 4:**
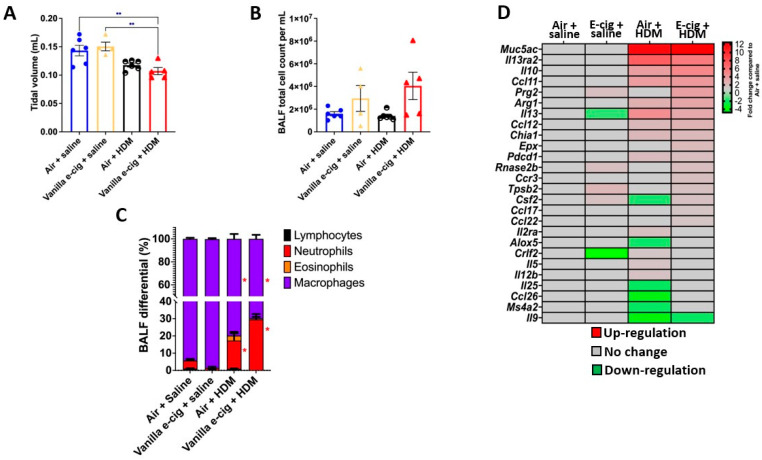
Prenatal exposures to vanilla-flavored e-cig aerosol aggravate house dust mite (HDM)-induced asthmatic responses in male mouse offspring. (**A**) Prenatal vanilla-flavored e-cig aerosol exposures + HDM significantly decreased lung tidal volume compared to baseline groups (prenatal air + saline and prenatal e-cig + saline. (**B**) Following HDM treatment, there was no significant difference in the BALF total cell count; however, (**C**) differential cytology analysis revealed that the HDM treatment (prenatal air + HDM & prenatal e-cig + HDM) resulted in a significant neutrophilic inflammatory response compared to baseline groups. Data are expressed as mean ± SEM. N = 4–6 male mice per group. ANOVA followed by a Tukey post hoc test. ** *p* < 0.05 compared to Vanilla e-cig + HDM group. * *p* < 0.05 compared to Air + Saline group. Circles represent individual results for mice in the in utero air group (blue = Air + saline and back and white = Air + HDM). Triangles represent individual results for mice in the in utero vanilla e-cig group (yellow = Vanilla e-cig + saline and red = Vanilla e-cig + HDM). (**D**) PCR array analysis showed that the HDM treatment significantly dysregulated the expression of genes associated with allergy and asthma. Red = up-regulation; green = down-regulation. Values are presented as fold-changes compared to respective air controls. |Fold-change| > 1.5 was considered significant. N = 4 male mice per group.

**Figure 5:**
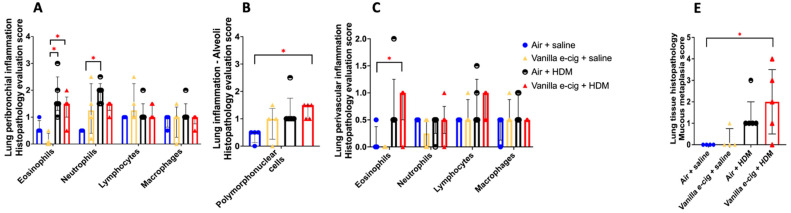

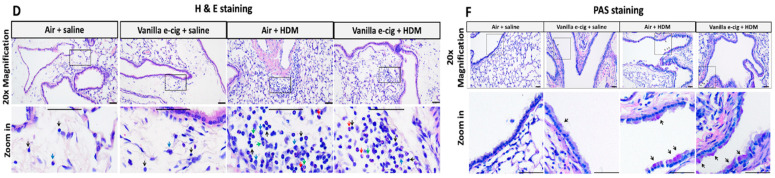
Prenatal exposures to vanilla-flavored e-cig aerosol + HDM treatment exacerbates lung mucous metaplasia. (**A**–**C**) Histopathological assessment of the lung tissue for peribronchial, alveoli and perivascular inflammation. (**D**) Representative H&E-stained lung slide for each group. All scale bars are 50 μm. Images show low numbers of perivascular and peribronchial leukocytes in the Air + saline and Vanilla e-cig + saline groups. Leukocyte numbers are increased in the Air + HDM and Vanilla e-cig + HDM groups. Blue arrows indicate macrophages; black arrows indicate lymphocytes; red arrows indicate eosinophils; and green arrows indicate neutrophils. (**E**) Histopathological assessment of lung mucous metaplasia. (**F**) Representative PAS-stained lung slides. All scale bars are 50 μm. No PAS-positive cells (purple stained cytoplasm) are observed in the control group (Air + saline), and rare scattered PAS-positive cells (arrows) are noticed in the vanilla e-cig + saline group. Minimal and mild increases of PAS-positive cells (arrows) are noticed in Air + HDM and Vanilla e-cig + HDM groups, respectively. Data are expressed as median ± interquartile range. The interquartile range shows the distribution of the data between the 25th and the 75th percentile. N = 4–5 male mice per group. The Kruskal–Wallis test was used to determine statistical differences. * *p* < 0.05.

Since the lung transcriptomes of newborn male mouse offspring were more affected by the in utero vanilla-flavored e-cig aerosol exposure than that of the female counterparts, in terms of both the number of dysregulated genes as well as the magnitude of the fold changes (Figure 1), we then assessed, in the 7-week-old male mouse offspring, whether the in utero e-cig aerosol exposure represents a significant baseline effect impacting responses to a common allergen later in life. Sub-groups of 4-week-old in utero-exposed male mouse offspring were treated with either saline or HDM once a week for three consecutive weeks, and lung function and pulmonary responses were examined at 7 weeks of age. Whole-body plethysmography results showed that the superimposition of the HDM treatment onto the in utero vanilla-flavored e-cig aerosol exposures significantly decreased the tidal volume of those juvenile male mice compared to the non-HDM exposed in utero air + saline and vanilla e-cig + saline counterparts (Figure 4A). Although there were no significant differences in the BALF total cell count between the groups (Figure 4B), the differential analysis of BALF cytology revealed that the percentage of neutrophils was significantly higher and that of macrophages significantly lower in both HDM treated groups compared to the respective saline groups (Figure 4C). The percentage of BALF neutrophils in the in utero vanilla-flavored e-cig aerosol + HDM male mouse offspring (29.5%) was almost double that of the in utero air + HDM exposed counterparts (16.5%) (Figure 4C). Further, at the lung tissue level, we observed increased peribronchial and perivascular eosinophils, as well as augmented alveolar polymorphonuclear cells, in the in utero vanilla-flavored e-cig aerosol + HDM male mouse offspring compared to the air group (Figure 5). In addition, the presence of lung mucous metaplasia was significantly augmented in the in utero e-cig + HDM exposed offspring compared to the air control group (Figure 5E). These results show that the lungs of the in utero-e-cig-exposed male mice showed abnormalities in terms of an increased presence of inflammation, whether peribronchial or perivascular, and mucus (Figure 5). At the molecular level, the evaluation of 84 genes related to asthma and allergy indicated that the lung transcriptomic baseline effect of the in utero vanilla-flavored e-cig aerosol exposure resulted in the up-regulation of four genes (*Prg2*, *Rnase2b*, *Tpsb2*, and *Csf2*, fold-change ranged from 1.7 to 2.2) and the down-regulation of two genes, *Il-13* and *Crlf2* (fold-change range −1.5 to −4.8) (in utero vanilla e-cig + saline versus in utero air + saline) (Figure 4D). Moreover, the in utero-exposed mice that received HDM in both groups exhibited dysregulation, mostly up-regulation, of several lung genes related to inflammation, thus supporting the cytology and histopathology results (Figure 4 and Figure 5). While 12 genes were up-regulated by the in utero air + HDM treatment, including *Muc5ac*, *Arg1*, *Il-13*, *Il-13ra2*, *Il-10*, and *Ccl12* (fold-change range 2.2 to 11.7), 17 genes were up-regulated in the mice exposed in utero to vanilla-flavored e-cig aerosol and treated with HDMs (fold-change range 1.5 to 12.5) (Figure 4D). In addition to the genes dysregulated by the HDM treatment (in utero air + HDM), the genes uniquely dysregulated by the e-cig aerosol exposure plus the HDM treatment included, *Prg2*, *Epx*, *Rnase2b*, *Ccr3*, and *Tpsb2* (fold-change range 1.7 to 3.6) (Figure 4D). The up-regulation of *Prg2*, *Rnase2b*, and *Tpsb2* was exclusively conserved in both in utero vanilla-flavored e-cig aerosol exposed groups (in utero vanilla e-cig + saline and in utero vanilla e-cig + HDM groups) (Figure 4D). Overall, at the physiological (Figure 4A), tissue (Figure 5), cellular (Figure 4C), and molecular (Figure 4D) levels, our lung data strongly suggest that in utero exposures to vanilla-flavored e-cig aerosol exacerbate HDM-induced asthmatic responses in juvenile male mouse offspring.

## 4. Discussion

Molecular abnormalities in developing lungs can result in mortality and morbidity, including marginal lung changes that can promote subsequent chronic lung diseases [39,40,41,42,43]. This highlights the importance of understanding the transcriptomic effects of in utero ENDS aerosol exposures on developing lungs. Here, vanillin, a flavoring chemical known to cause DNA damage [29,30], was examined for its effects on the lung transcriptome at birth and later for predisposing offspring to asthma development during childhood. In addition, vanilla-flavored e-cig aerosols have previously been shown to affect lung function and cause sensory irritation in the upper respiratory tract of mice [44,45]. We conducted in utero exposure of mice to vanilla-flavored e-cig aerosols and challenged male mouse offspring with HDMs starting at 4 weeks of age (Figure 1, Figure 2, Figure 3, Figure 4 and Figure 5). Overall, we found that the RNA sequencing of whole-lung homogenates of 1-day-old in utero-exposed mouse offspring showed altered transcriptomic responses imprinted by in utero vanilla-flavored e-cig aerosol on the developing lungs (Figure 1). These molecular signatures included the down-regulation of six protein-coding genes (*Adamts1*; *Agxt*; *Errfl1*; *Slk1*; *Per1*; *Zfp791*) common to both sexes, with male offspring displaying greater fold changes for all six genes (Figure 1). *Adamts1* is a metalloendopeptidase that is involved in tissue morphogenesis [46], which deficiency in mice leads, among others, to growth delay [47]. This gene, also associated with extracellular matrix formation, was previously identified in the developing mouse lung [48]. *Errfl1* is an inhibitor of the epidermal growth factor receptor (EGFR), implicated in cell proliferation [49], while *Slk1* was previously shown, in yeast, to affect cell morphogenesis and proliferation processes [50]. *Per1* is a member of the core Clock oscillatory network, which plays a key role in circadian rhythm [51]. Clock oscillation, as evidenced by *Per1* gene expression in the embryonic rodent lung, has been previously demonstrated [51]. Clock-related responses are highly shaped by their environment, and thus the down-regulation of *Per1* suggests insufficient clock adjustment in the lungs of the in utero e-cig exposed mouse offspring at birth, which in turn, may support the concept of in utero vanilla-flavored e-cig aerosol exposures affecting fetal lung programming [51]. Overall, common down-regulated genes in both male and female mouse offspring exposed in utero to vanilla-flavored e-cig aerosols suggest that the lungs may adapt to this environmental insult by reducing the expression of protein-coding genes involved in tissue/cell morphogenesis and cell proliferation processes, as well as in Clock biology. In total, the in utero vanilla-flavored e-cig aerosol exposure dysregulated 88 and 65 genes in male and female offspring, respectively, with the majority of the transcriptomic alterations being sex-specific (Figure 1). Further, while several protein-coding genes were dysregulated, the expressions of numerous lincRNA (e.g., Gm20186 and Gm20507) were also impaired (Figure 1). Although the roles of lincRNA in lung development are currently unknown, it has been reported that lincRNA is involved in the regulation of gene expression occurring through interactions with chromatin-modifying complexes and, thus, via epigenetic mechanisms [35]. Hence, in the context of the developmental origin of health and disease and fetal lung programming, these findings, related to the dysregulation of lincRNA by in utero e-cig aerosol exposures, are novel and intriguing. Our data also demonstrate that in utero exposures to vanilla-flavored e-cig aerosol alter the developing mouse lung transcriptome at birth, with dysregulation of genes focused on immune (e.g., *Cd5*, *Cd6*, and *Ccr9*) and oxidative stress (e.g., *Map2k6*, *Maff*, and *Fos*) responses, in male and female mouse offspring, respectively (Figure 1, Figure 2 and Figure 3). Taken together, these data provide valuable insights into the molecular mechanisms by which vanilla-flavored e-cig aerosol could cause fetal lung programming leading to an increased risk of having poor lung function and developing lung diseases later in life.

Lung development is orchestrated by a complex yet precise temporal and spatial expression of genes [52,53]. The development of a healthy and efficient lung requires at least two distinct key processes, branching morphogenesis and epithelial differentiation, which include the formation of the conducting airways and the alveolar space [43,54,55]. Delta-like 1 homolog (*Dlk1*) plays a significant role in the Delta–Notch signaling pathway, which is essential for lineage commitment and branching morphogenesis during lung development [56]. Thus, the expression of DLK1 is elevated during embryonic development [56,57]. In our study, male mouse offspring exposed in utero to vanilla e-cig aerosol at birth exhibited decreased gene expression of *Dlk1* by 2.2-fold compared to the respective air control group (Figure 1C). Since it was previously shown that DLK1 marks the areas of the lungs that are implicated in branching morphogenesis [56], our data regarding male offspring suggest that in utero exposures to vanilla e-cig aerosol, by decreasing the expression of *Dlk1*, which is vital during embryogenesis, can impact branching morphogenesis. Given that we only focused on the effects on the lung transcriptome and not on the lung anatomy/architecture in this study, more research is needed to investigate whether this molecular change associated with branching morphogenesis translates into lung branching structural changes. Further, *Septin-2* has been shown to be a vital protein-coding gene implicated in controlling the barrier function of the airway epithelium [58]. Indeed, as barrier function increases, including in reaction to shear stress, the expression of *Septin-2* increases as well [58]. Enhanced barrier function will impact, among others, tight junction paracellular barriers and, thus, subsequently cell signaling. In our in vivo model, male mouse offspring exposed to vanilla-flavored e-cig aerosol in utero displayed increased lung gene expression of *Septin-2* at birth by 2.8-fold (Figure 1). This suggests that the in utero vanilla-flavored e-cig aerosol exposure increased the airway epithelium barrier function, which may reduce cell signaling during critical stages of lung organogenesis, where highly coordinated temporal expression of genes is vital for the normal structural and functional development of the lungs.

Another essential element of lung development includes the production of pulmonary surfactant. The protein-coding gene ATP-binding cassette transporter A1 (*Abca1*), which is involved in reverse cholesterol and phospholipid transport, plays a significant role in lung development [59,60]. In *Abca1* knockout mice, impaired lung lipid metabolism and homeostasis were evidenced by the accumulation of surfactant within the alveolar spaces in addition to elevated levels of lung tissue cholesterol [60]. In our study, in the male mouse offspring exposed in utero to vanilla e-cig aerosol, the gene expression of *Abca1* at birth was down-regulated by 1.9-fold compared to the respective air control group (Figure 1C). These data suggest that in male mouse offspring, in utero exposures to vanilla-flavored e-cig aerosol may alter the lipid/cholesterol alveolar microenvironment, which could affect the physiological characteristics of pulmonary surfactants, indispensable to normal lung function [60,61]. Moreover, the Nuclear Factor of Activated T cells calcineurin-dependent 3 (*Nfatc3*) is a transcription factor playing a key role in the synthesis of surfactants during lung development since it is involved in the direct activation of *Sftpa*, *Sftpb*, and *Sftpc*, three of the main surfactant proteins [52]. Transcriptomic alterations of genes regulating surfactant homeostasis at birth could have detrimental consequences on the lungs, including on lung function at birth and in early post-natal life [42]. In this study, in female mouse offspring, the expression of stearoyl-coenzyme A desaturase 1 (*Scd1*) gene, which is involved in pulmonary surfactant phospholipid metabolism [52], was down-regulated −1.9-fold compared to air controls (Figure 1D). The *Scd1* gene was also significantly down-regulated in the lung epithelial cells of neonatal mice deficient in *Cnb1*, a gene involved in NFAT signaling [52]. Taken together, these data indicate that both male and female mouse offspring exposed in utero to vanilla-flavored e-cig aerosol exhibit dysregulated expression of key genes directly involved with the production and maintenance of pulmonary surfactant.

Inflammation and oxidative stress are two types of cellular responses that can negatively affect lung development [62,63]. NF-kB is a transcription factor that plays several roles in lung development, including cell proliferation, in addition to having pro- and anti-inflammatory functions [62,64]. Indeed, inflammation can affect normal lung development, particularly alveologenesis [62]. While NF-kB is highly expressed at the beginning of the alveolar stage of lung development in mice, where it is involved in epithelial cell proliferation and differentiation, it was shown that inflammation impairs alveologenesis in human and animal studies [62,65,66]. Overall, increasing evidence reveals that NF-kB plays vitally important roles during the late saccular and early alveolar stages [62,67]. This corresponds to the period at which we evaluated the developing lungs in our mouse offspring, since at 1-day of age, mouse lungs are in the late saccular stage. Interestingly, in the male mouse offspring exposed in utero to vanilla-flavored e-cig aerosol, the canonical pathways associated with the dysregulated genes included NF-kB signaling (Figure 2). This suggests that in utero exposure may affect the genes involved in the activation or suppression of inflammatory responses, which in turn may affect lung maturation processes. Moreover, *Nrf2* is a transcription factor that is regulated by the redox state of the cell, and during lung development, it plays key roles in the control of immune mediators [63]. *Nrf2* contributes to the anti-oxidant responses by inducing the expression of cytoprotective genes and also has anti-inflammatory properties [63,68]. Previous studies conducted on *Nrf2-*deficient mice revealed impaired alveolarization, including the hindered progression from the saccular to the alveolar stages following hyperoxia [69,70,71]. Similarly, in neonatal mice, increased levels of *Nrf2* reduce hypoalveolarization following oxidant exposures [72,73]. These studies indicate that Nrf2 is essential to the developing lungs, including at birth. In our study, in female mouse offspring, we found that the dysregulated genes associated with the *Nrf2* signaling pathway were down-regulated and included *Maff* (fold-change = −1.9) and *Fos* (fold-change = −2.2) (Figure 1D and Figure 3). This suggests that the lungs of female mouse offspring exposed in utero to vanilla-flavored e-cig aerosols may be prone to lung structural effects, including enlarged airspaces, indicative of hypoalveolarization, as seen in pulmonary bronchial dysplasia. Although in this study, we did not investigate the effects of in utero ENDS exposure on alveolarization processes, these results are consistent with those in our previous study that showed that mouse offspring exposed in utero to cinnamon-flavored e-cig aerosol had increased mean linear intercept values, a measure of airspaces in the lungs, at 4 weeks of age, compared to the air control group [22].

Canonical *Wnt* is the leading signaling pathway controlling lung development [54]. We previously showed using PCR arrays, a qRT-PCR-based assay, that pre-conception and prenatal exposures to cinnamon-flavored e-cig aerosols containing 36 mg/mL of freebase nicotine altered the expression of 84 genes related to *Wnt* signaling in the lungs of mouse offspring at birth [22]. In a follow-up study, in utero exposures to this same e-cig aerosol dysregulated the expression of 39 *Wnt* signaling genes in male mice at post-natal day 5, whereas 61 *Wnt*-related genes were dysregulated in 5-day-old females [28]. In those studies, the canonical pathways associated with the dysregulated genes included Wnt/β-catenin signaling, TGF-β signaling, the role of Nuclear Factor of Activated T cells (NFAT) in the regulation of immune responses, and the planar cell polarity (PCP) pathway [22,28]. In this study, RNA sequencing revealed that 88 genes in male offspring and 65 genes in female offspring were dysregulated at birth following in utero exposures to vanilla-flavored e-cig aerosols containing 18 mg/mL of freebase nicotine, and dysregulated canonical pathways included the role of NFAT in the regulation of immune responses and NRF2-mediated oxidative stress responses (Figure 2 and Figure 3). Thus, dysregulated genes associated with the role of NFAT in the regulation of immune responses were common to all of these in utero e-cig aerosol exposures. This occurred even though qRT-PCR and RNA sequencing are two different technologies that provide information on the same biological outcome, gene expression [74]. While RNA sequencing clearly has the advantage of being an untargeted approach, enabling the detection of dysregulated genes in a global manner, qRT-PCR, which is a more limited targeted approach, is usually considered the gold standard for gene expression results [74,75,76,77]. Thus, although our published gene expression results [22,28] and the data from this current study (Figure 1) were obtained under different e-cig aerosol exposure conditions, i.e., different flavors (cinnamon vs. vanilla), nicotine concentrations (36 vs. 18 mg/mL), as well as, most importantly, the gene expression methods used (PCR arrays vs. RNA sequencing), the data show that in utero exposures to flavored e-cig aerosols containing freebase nicotine can affect the lung transcriptome, particularly protein-coding genes related to the nuclear factor of activated T cells and immune responses, in mouse offspring at birth and in early life.

Moreover, in the lungs of healthy mice, the transcription factor *Klf15* is up-regulated by 5.3-fold at embryonic day 16, whereas *Fos* is up-regulated 5.9-fold at embryonic days 18–19 [43,78]. In our study, in the male mouse offspring exposed in utero to vanilla e-cig aerosol, the gene expression of *Klf15* at birth (day 21) was down-regulated by 1.9-fold, while in female mouse offspring, *Fos* was down-regulated by 2.2-fold, compared to the respective air control group (Figure 1C,D). The expression of *Klf15* is induced by the glucocorticoid receptor, and this gene plays a fundamental role in lipid and glucose metabolism, as well as in regulating airway contractility [79,80]; meanwhile, *Fos* is involved with cytodifferentiation and apoptosis, with downstream effects seen in lung innate immunity [43]. Together, these results suggest that in utero exposures to vanilla-flavored e-cig aerosol can dysregulate the expression of genes involved in fetal lung maturation as well as lung function. Further, *Atp6v0d2* is a protein-coding gene for a subunit of vacuolar ATPase, a proton pump located, among other cells, on macrophages [81]. Gene expression for *Atp6v0d2* is elevated in asthmatic lungs and in mice treated with ovalbumin [81]. In our study, in the female mouse offspring exposed in utero to vanilla e-cig aerosol, the gene expression of *Atp6v0d2* at birth was up-regulated by 2.2-fold compared to the respective air control group (Figure 1D). This implies that in utero exposures to vanilla-flavored e-cig aerosol may predispose the lungs of the mouse offspring to an asthmatic milieu.

Based on the developmental origin of health and disease, in utero exposure to environmental pollutants can alter the trajectory of heath by increasing the susceptibility to chronic diseases later in life [14,15,16,17,18,82]. It is well-documented that the lungs are sensitive to ‘fetal programming’, as epidemiological and experimental evidence shows that in utero exposure to cigarette smoke, second-hand smoke, and ENDS aerosols can lead to adverse pulmonary responses in the offspring, including increased risk of developing asthma [11,12,13,22,23,82,83]. Asthma is a chronic lung disease affecting 339 million individuals worldwide, including children [84]. The prevalence of asthma during childhood is greater in boys than in girls, while the tendencies are opposite in adolescence and in adulthood, where females are more affected than males [84]. Asthma mainly affects the lower respiratory tract, and symptoms include bronchospasm, airflow limitations, increased mucus production, and lung inflammation [81,85]. The cellular infiltrates of leukocytes in the lungs are mainly driven by neutrophils, in severe steroid-resistant cases, or eosinophils, in allergy or non-allergy-related cases [81,85,86,87]. Additionally, the presence of Th2-mediated cytokines is a hallmark of asthmatic responses related to eosinophilic inflammation [81,85]. In the present study, we found that in utero exposures to vanilla-flavored e-cig aerosols containing 18 mg/mL of nicotine increased asthmatic responses in HDM-treated juvenile male mouse offspring compared to the Air + HDM exposed controls (Figure 4). This was evidenced by a decrease in the tidal volume as well as by an increase in BALF neutrophilic inflammation observed at the molecular, cellular and tissue levels (Figure 4 and Figure 5). Overall, these results suggest that in utero e-cig aerosol exposure results in a fragile lung that could exaggerate the responses to subsequent allergen exposure.

While we saw no significant changes in the baseline effects of in utero e-cig aerosol exposures (e-cig + saline) on tidal volume and BALF inflammation compared to results for air counterparts at 7 weeks of age, molecular alterations were seen in the lungs, with the dysregulation of 6 genes (Figure 4). Of those dysregulated genes, four were associated with immune-mediated inflammatory diseases (*Csf2*; *Crlf2*; *Rnase2b*; *Tpsb2*) and one with airway hyperresponsiveness (*Prg2*), while *Il-13* was associated with both of those pathways (Figure 4D). This highlights that in utero vanilla-flavored e-cig aerosol exposures can induce long-term molecular effects on the lungs that persist at least until 7 weeks of age. In another study, we found that molecular signatures induced by in utero mint-flavored JUUL aerosols on the lungs of male mouse offspring persisted until 11 weeks of age and consisted of 16 genes, which were mainly down-regulated [23]. Further, it was recently shown that in utero exposures to unflavored e-cig aerosols, with or without nicotine, affect lung structure and function in adult 5-month-old offspring [83]. Together, these results clearly demonstrate that long-term pulmonary sequelae are associated with a single in utero e-cig aerosol exposure in mouse models.

Moreover, mice exposed in utero to vanilla-flavored e-cig aerosols followed by a second challenge to an allergen later in life exhibited decreased tidal volume, increased BALF neutrophilic inflammation, and increased lung peribronchial and perivascular eosinophils (Figure 4 and Figure 5). These results were further supported by the up-regulation of 17 genes associated with allergy and asthma (Figure 4D). The overexpression of *Muc5ac*, which was up-regulated by 12.5-fold compared to the air + saline group (Figure 4D), and further supported by the significantly increased presence of mucous metaplasia in the lungs, as evidenced by the histopathology evaluation (Figure 5D), implies the overproduction of mucus in the airways, which is known to negatively affect the mucociliary clearance of pathogens and also cause a decline in lung function [88]. Hence, the decreased tidal volume we observed in the in utero-exposed mice treated with HDM (Figure 4A) could be due to the increased presence of mucus in the lungs as well as the increased percentage of leukocytes (Figure 4 and Figure 5). In contrast, the mice exposed in utero to air and treated with HDM exhibited an increase in the BALF percentage of neutrophils with an up-regulation of 12 genes (Figure 4C). Thus, these data indicate that in utero exposures to vanilla-flavored e-cig aerosol exacerbated asthmatic-induced pulmonary responses in our mouse model.

In addition, Th2 cytokines, present during eosinophilic inflammation, can induce the expression of arginase in macrophages [89]. Arginase 1 (*Arg1*), a nitric oxide substrate that regulates bronchomotor tone and inflammation, plays a significant role in asthma development [85]. Additionally, *Arg1* expression is increased at the gene and protein levels in the lungs and serum of HDM-induced asthmatic mice [90]. In our study, 7-week-old male offspring exposed in utero to vanilla-flavored e-cig aerosol and subsequently exposed to HDM exhibited a 3.2-fold increase for this gene (Figure 4). Overall, these results imply that in utero exposures to vanilla-flavored e-cig aerosol can dysregulate the expression of *Arg1* in the lungs following exposure to an allergen, e.g., HDM. This corresponds with the pathogenesis of asthma, as seen in other mouse models [85,90].

We previously reported that in utero exposures to mint-flavored JUUL aerosols exacerbate HDM-induced asthma in 11-week-old mouse offspring [23]. Although the asthmatic responses were more pronounced in the in utero JUUL-exposed females compared to the males, we found that 33 asthma- and allergy-related genes were significantly up-regulated in the in utero JUUL-exposed males treated with HDM in comparison to the air + HDM controls [23]. Of those 33 up-regulated genes, 10 are commonly found in our in utero vanilla-flavored e-cig aerosol + HDM-exposed male mice. These genes include *Ccl12*, *Rnase2b*, *Il10*, *Ccl11*, *Ccr3*, *Prg2*, *Epx*, *Tpsb2*, *Arg1*, and *Csf2*, with fold-changes ranging from 1.5 to 4.4 and from 1.7 to 4.9 for the in utero JUUL and vanilla e-cig aerosol-exposed mice, respectively [23] (Figure 4D). With only the exceptions of *Ccl12* and *Rnase2b*, all commonly dysregulated genes were up-regulated to a greater extent in the male mouse offspring that had been exposed in utero to vanilla flavored e-cig aerosol compared to the males exposed to in utero to JUUL [23] (Figure 4D). For instance, *Il-10*, a Th2-derived cytokine with both pro-and anti-inflammatory properties [91,92,93], was up-regulated two-fold in the in utero mint-flavored JUUL + HDM males, while this gene was up-regulated 4.9-fold in the in utero vanilla-flavored e-cig aerosol + HDM males [23] (Figure 4D). The greater impact of the baseline in utero ENDS aerosol exposure effect could be due to the flavoring chemicals found in the aerosols, as well as to the nicotine form (freebase vs. salt); in addition to the fact that in our vanilla-flavored e-cig aerosols study, the male mouse offspring were challenged with HDM at 4 weeks of age, and the asthmatic responses evaluated at 7 weeks of age, whereas in the mint-flavored JUUL study, the males were challenged at 8 weeks of age, with the outcomes assessed at 11 weeks of age. This suggests a larger influence of the baseline in utero ENDS aerosol exposure effect on lung responses in childhood rather than in adulthood. Additionally, we previously reported on the increased expression of *Il-10* following in utero exposures to mint-flavored JUUL aerosols in the lungs of the mouse offspring at birth, as well as following HDM treatment at 11 weeks of age [23]. These data also correlated with decreased methylation of the *Il10ra* gene in HDM treated in utero JUUL-exposed mouse offspring at 11 weeks of age [23]. Globally, these results [23] are in line with our current data showing up-regulation of *Il-10* in the lungs of male mouse offspring exposed in utero to vanilla-flavored e-cig aerosols (Figure 4D) and point in the direction that in utero ENDS aerosol exposures may impact the expression of *Il-10*, a key player in asthma-related pulmonary responses. Whether the in utero e-cig aerosol exposure associated with increased *Il-10* gene expression following an allergen challenge occurs through epigenetic mechanisms is still unclear and requires further investigation. Overall, our published data [22,23,28], as well as the results from the current study (Figure 4 and Figure 5), strongly suggest that in utero exposures to ENDS aerosols may have lasting effects on the lungs of the offspring, including increasing the susceptibility to a heightened response following exposure to an allergen later in life.

## 5. Limitations

Although the scope of the current study was narrow and aimed at deepening our knowledge of the key genes and pathways that are dysregulated by prenatal vanilla-flavored e-cig aerosol exposures on the mouse developing lungs at birth to subsequently help better understand the heightened responses following an allergen exposure later in life, this study has a few limitations. First, we exposed the dams to an e-cig aerosol composed of PG, VG, nicotine, and vanilla flavoring chemicals; therefore, the current dataset does not allow for discrimination of the main e-liquid ingredient driving the responses. However, since young adults who vape are most likely simultaneously exposed to PG, VG, nicotine, and flavoring chemicals in the e-cig aerosols, our study is representative of ‘real-world’ exposure scenarios. This study also does not us allow to compare the toxicity of various flavored e-cig aerosols, as we only used mice exposed in utero to air for controls. Second, at birth, we only evaluated the lung transcriptomic alterations caused by prenatal exposure via RNA sequencing, and no other pulmonary outcomes were assessed. While the expression of specific genes was not validated by qRT-PCR, RNA sequencing plus appropriate bioinformatics analyses are reliable methods for transcriptomic assessment [74,75,76,77]. Further, correlations between molecular changes and lung structural or physiological effects at birth cannot be made in this study. Third, although the lung transcriptomic data impacted by in utero e-cig aerosol at birth represent a snapshot in time, more research is needed to evaluate how lung gene expression changes over time and whether the effect is e-cig aerosol dose-dependent. Fourth, only the male mouse offspring were exposed to an allergen later in life; hence, sex-specific differences with regard to how prenatal e-cig aerosol exposures can increase the susceptibility to develop asthma could not be established in this study. Nonetheless, this small study is informative and provides novel data, as we are the first to describe transcriptomic alterations, evaluated through RNA sequencing, in the developing mouse lungs at birth caused by in utero vanilla-flavored e-cig aerosol exposures. Overall, these results help lay the groundwork and offer direction for future studies that will include proteomic, lipidomic, structural and physiological assessments of the lungs following in utero exposures to e-cig aerosols.

## 6. Conclusions

Previous studies have shown that in utero ENDS aerosol exposures can affect the developing lungs in several ways, including effects seen at the structural [22], functional [10,28,83], biochemical [9,94], and molecular levels [9,22,23,28,94]. While the underlying mechanisms by which in utero exposures to environmental pollutants affect the health trajectory of the lung are mainly unknown, commonly accepted explanations include epigenetic modifications and gene expression alterations in the developing fetal lung [82,95]. Thus, here we focused on investigating the transcriptomic effects of in utero exposures to e-cig aerosol on the lungs at birth. We identified signaling pathways, including NFAT, NF-kB, and NRF2, that are altered by in utero e-cig aerosol exposures. Understanding the gene networks and key molecular pathways of lung organogenesis that are altered by in utero exposures to e-cig aerosols is essential to develop strategies related to the prevention and treatment of in utero e-cig exposures associated with lung diseases. This study also provides evidence supporting that the inhalation of e-cig aerosols during pregnancy is detrimental to the respiratory health of the offspring and may increase asthma severity in juvenile male mice. Thus, our data indicate that in utero exposures to e-cig aerosols may represent another environmental risk factor implicated in the developmental origin of health and disease, particularly asthma. More research is still needed to elucidate the specific mechanisms by which prenatal exposures to e-cig aerosols affect lung responses throughout the lifespan.

## Figures and Tables

**Table 1:** In utero vanilla-flavored ENDS aerosols exposure characterization.

Parameters	Session 1		Session 2	
	Air	Vanilla E-Cig Aerosol	Air	Vanilla E-Cig Aerosol
Aerosol concentration (mg/puff)	-	0.13 ± 0.04	-	0.16 ± 0.07
Temperature (°C)	25.0 ± 2.6	21.7 ± 1.1	26.0 ± 2.4	23.2 ± 1.5
Humidity (%RH)	49.5 ± 9.3	81.9 ± 11.5	51.0 ± 0.1	47.0 ± 0.2

Data are expressed as mean ± standard deviation (SD).

## Data Availability

Data are available upon reasonable request.
